# Psychosocial Dysfunction in Major Depressive Disorder—Rationale, Design, and Characteristics of the Cognitive and Emotional Recovery Training Program for Depression (CERT-D)

**DOI:** 10.3389/fpsyt.2017.00280

**Published:** 2017-12-12

**Authors:** Matthew James Knight, Bernhard T. Baune

**Affiliations:** ^1^Discipline of Psychiatry, University of Adelaide, Adelaide, SA, Australia

**Keywords:** depression, cognitive training, cognitive remediation, psychosocial functioning, social cognition, emotion processing

## Abstract

**Introduction:**

Psychosocial dysfunction is associated with poor longitudinal course of depression and is not sufficiently addressed by existing pharmaceutical or psychological treatments. The aim of the current study is to evaluate the efficacy of a novel intervention designed to improve psychosocial function in depressed individuals. Impaired cognition, emotion processing, and social cognition appear to underlie (i.e., cause) psychosocial dysfunction in depression. The current treatment will target functioning in these domains (i.e., cognition, emotion, social cognition) with repeated training tasks, following the rationale that therapeutic benefits will arise in psychosocial functioning. It is expected that personalizing treatment by participants’ baseline functioning will enhance clinical efficacy, by comparison with standard treatment in which baseline functioning is not considered.

**Methods:**

The study is a randomized, controlled treatment (RCT), in which the efficacy of a personalized and standard intervention will be compared. Sixteen treatment sessions will be administered over an 8-week period. These treatments are designed to improve cognition, emotion processing and social cognition. Assessments of psychosocial functioning, as well as a number of secondary outcomes, will occur at baseline, 4 weeks (mid-RCT), 8 weeks (end of RCT), and in the observational period at baseline (week 9) and 3 and 6 months post-RCT. Recruitment will commence in July 2017, including subjects diagnosed with major depressive disorder according to DSM-IV-TR criteria.

**Discussion:**

This research will provide new insight into the roles of cognition, emotion processing, and social cognition in psychosocial dysfunction in depression. In addition, the relative clinical efficacy of personalized versus standard treatment approaches will be assessed.

**Ethics and dissemination:**

This study has been approved by the human research ethics committees of the Royal Adelaide Hospital and the University of Adelaide (ethics code: R20170611). The study has been registered with the Australia and New Zealand Clinical Trials Registry Registration number: ACTRN12617000899347, web link: http://www.anzctr.org.au/Trial/Registration/TrialReview.aspx?ACTRN=12617000899347p. The results of the current study will be published in academic journals following completion of recruitment in 2019. Data will be owned and retained by the University of Adelaide, with access restricted to the research team responsible for the study.

## Introduction

Depression is the leading cause of mental illness worldwide, affecting approximately 322 million individuals (1). The illness is characterized by prolonged negative mood, anhedonia, and impaired cognition. Depressed individuals also demonstrate significantly impaired psychosocial function, indicated by diminished organizational, occupational, and social ability (2, 3). In addition to the substantial burden of depression on the daily lives of individuals (4) depression impacts on a societal level by reducing occupational productivity (5, 6). Established therapies (e.g., psychotherapy, CBT) and pharmaceutical treatments are costly and lead to high rates of recurrence in the long term (6). As a consequence, there is a clear need to develop alternative and/or complimentary treatments for depression.

It is possible that existing treatments for depression underperform because they do not sufficiently address psychosocial functioning (2, 7). Psychosocial function can be defined on a micro level as our day-to-day ability to contend with environmental and social tasks (e.g., maintaining work and relationships), and on a macro level as the pursuit of significant life outcomes (e.g., self-actualization) (7). Previous work has operationalized these dimensions, such that quantitative and psychometrically valid assessments of psychosocial impairment have been established (7–9). Psychosocial functioning appears to be related to a number of other functional outcomes (e.g., positive future outlook, ability to derive pleasure from life events), suggesting that psychosocial function contributes to mental health in general (10, 11). Psychosocial dysfunction is prevalent in depressed individuals and does not appear to improve even in patients who are symptomatically recovered (3). Ongoing psychosocial dysfunction may lead to recurrent episodes of depression, as impaired functioning negatively interacts with cognitive and emotional vulnerability in previously depressed individuals (2, 12–14).

Existing evidence indicates that cognitive, emotional, and social cognitive factors underlie deficits in psychosocial functioning (11, 13–16). The proposed study follows this model, stipulating that improvements in cognition, emotion, and social cognition should flow on to psychosocial functioning. Given the importance of this model in the current study, the relationship between the three underlying domains and psychosocial functioning will be explored.

### Cognitive Impairment

According to DSM-5 criteria the cognitive symptoms of major depressive disorder (MDD) are impaired decision making, management of attention, and coordination and maintenance of information in working memory (17). Research has also identified deficits in verbal ability (18), visuospatial processing (19), and psychomotor speed (19). The finding that cognitive deficits occur across multiple domains suggests that depression interferes with underlying cognitive faculties, and with the coordination of cognitive subsystems, rather than interfering a specific modality of cognition. Our cognitive abilities are crucial to daily functioning, and to the severity of several cognitive difficulties associated with depression (e.g., hyper-sensitivity to negative feedback). Cognitive treatments target improvement of cold cognition, with the rationale that increasing functioning will benefit performance in hot cognitive tasks, and hence improve experience of everyday life, psychosocial functioning, and day-to-day functioning. In addition, cognitive impairments may mutually interact with emotional and social factors to maintain or exacerbate depression, or lead to recurrent episodes (14, 16).

Psychosocial and cognitive impairments in depression and in other illnesses are associated with elevated levels of several inflammatory cytokines (20, 21). Research by the Baune group (22, 23) demonstrated that IL-8, IL-1beta, and tumor necrosis factor (TNF) are associated with memory, processing speed and motor function in the elderly. In addition, inflammatory C-reactive protein (CRP) may also be associated with depression symptoms (24), and the neurotransmitters 5HT, DA, and NE appear to be related to psychological stress (25). Investigating biomarkers for psychosocial functioning and cognitive dysfunction presents a valuable area for research, as there is potential for developing objective measurements of dysfunction predisposition, for improving our understanding of the neurological mechanisms of depression and associated poor psychosocial functioning (e.g., neurotrophic theory), and for improving pharmacological treatments that address not only symptoms of depression but also psychosocial functioning and workplace functioning.

A large body of work has investigated the selective treatment of impaired cognition in depression with cognitive remediation programs (2, 5, 26), which typically involve repeated completion of cognitive tasks over several weeks. The results of such programs generally reveal that patients improve on measures of executive, visuospatial, and verbal function (2). These results support the theory that repeated activation of brain regions *via* cognitive treatment increases neuroplasticity and improves neural function (27). Although cognitive gains following remediation programs are relatively consistent, the transfer of this benefit to occupational function, resilience, and psychosocial functioning are not well established (15). It is possible that transfer of cognitive skills does not reliably occur because remediation programs do not also address impaired social cognitive skills and emotional processing, which may negatively interact with cognition on depression outcomes. The following paragraphs discuss the role of emotion processing and social cognition in MDD, and highlight how an integrated and personalized treatment approach may be critical to maximizing treatment outcomes.

### Emotion Processing

Our experience of emotion is fundamentally linked to cognition. Evidence for this link is demonstrated at a neurological level by overlap in activation patterns of cognitive processes and emotional experience (28–30). The phenomenological parallels of emotion and cognition are consistent with cognitive models of depression (31), which stipulate that interplay between cognitive vulnerability and negative emotion both lead to and sustain MDD. Specifically, cognitive models stipulate that attention and memory systems are biased to focus on negative information, suppress adaptive coping strategies (e.g., flexibility), and encourage maladaptive strategies (e.g., rumination) (32). The critical importance of emotion processing in coping and information processing supports the importance of this factor in determining overall psychosocial functioning (15).

The close overlap between emotion and cognition suggests that cognitive remediation should not neglect the role of emotion in treatment programs (33). Previous work in cognitive–emotional treatment has shown promising results in the implementation of working memory tasks with valenced stimuli (34, 35). Iacoviello et al. (35) conducted a study comparing the benefit of an integrated cognitive–emotional treatment task with that of a pure cold cognitive treatment task for subjects with MDD. The results showed that the integrated cognitive-emotional training resulted in greater reduction in depression symptoms and negative self-referential biases. By contrast, both the cold cognition and integrated tasks resulted in similar gains in attention and working memory performance. These findings are consistent with the notion that cognition and emotion are closely linked, and imply that an integrated treatment approach may result in broader transfer of therapeutic benefit.

Recent work by Wu et al. (36) suggests that dysfunctional emotion processing may play a crucial role in the development and maintenance of geriatric depression. The authors evaluated the positive effect of reminiscence therapy, in which older female patients with depression symptoms verbally recounted earlier life experiences by viewing pictures of past events. A clinical nurse encouraged the patient to recount these memories from different perspectives, and highlighted positive comparisons with the patients’ current lives. Posttreatment anxiety and depression symptoms were reduced, suggesting that encouraging flexible and positive retrieval of episodic memory may attenuate negative emotion processing. It is possible that emotional dysregulation may play a greater role in geriatric depression than in standard MDD, as the elderly often lack sufficient social support and experience rapid physiological decline (36). Taken together, these findings suggest that older subjects in the current treatment may benefit from emotion processing training tasks focused on redressing negative social biases and negative self-evaluations.

### Social Cognition

Social cognition refers to the perception, identification, and interpretation of social information in interpersonal interaction (16). Maintaining function in this domain involves incorporating information from a range of social cues including prosody, facial expression, body language, verbal content, and theory of mind. Research has identified that social cognition may be impaired in individuals with depression, though the social cognitive deficit is less severe than in other psychiatric illnesses (e.g., schizophrenia) (37, 38). However, social cognition deficits in depression should not be overlooked, as issues with social interaction are associated with suicidality (39), and with severity of depression symptoms (40, 41).

Given the complexity of social interactions, it stands to reason that cognitive functions (e.g., attention, processing speed) are crucial in maintaining fluid and adaptive social ability. Likewise, emotional recognition and bias play an important role in identification and perception of social information. Impaired emotion recognition may cause incorrect or biased assessment of social interaction, which may exacerbate depressed mood and lead to further negative social interactions (16). In turn, negative social experiences may lead to subsequent avoidance of interpersonal interactions, further enhancing feelings of isolation and impaired mood. The interplay between social cognition, emotion, and general cognition further highlights the need for an integrated treatment approach. In addition, the broad and inter-related deficits associated with social cognitive issues suggest this factor contributes substantially to psychosocial functioning. This being said, the link between social cognition and psychosocial functioning has only recently been empirically investigated (16), and pilot data from our group suggest this relationship; supporting the need for the current research.

Social cognition is typically evaluated by one’s ability to read facial emotions (42). Depressed persons are typically impaired in facial affect recognition, in part due to a tendency to negatively interpret facial emotions (43). A plausible explanation is that impaired attention and emotional interpretation may cause depressed individuals to focus on mood-congruent (i.e., negative) features of facial affect. Other studies of social cognition employ videos of social interactions, which are intended to be more naturalistic and contain more dynamic social features (i.e., body language, prosody, verbal information) (44, 45). These tasks emphasize theory of mind, as reliance on syntactic and visual information alone is insufficient to detect nuanced social interactions (e.g., sarcasm). Social cognition can also be measured with prosody tasks, in which several syntactically identical sentences are presented with different emotional intonations.

The current treatment will use an integrated approach in which cognitive remediation and cognitive training techniques are employed with cognitive, emotional, and social cognitive stimuli. While traditional cognitive training has been shown to increase psychosocial functioning (46, 47), it is expected that the addition of emotional and social training domains will extend and enhance this effect. Similar techniques have been employed to address emotional and social impairment in other psychiatric illnesses, including bipolar (48) and schizophrenia (49). The results of these studies suggest that social and emotional training may provide benefits in domains of daily life (e.g., occupational functioning). The clinical efficacy of these strategies in treating major depression has not yet been evaluated and is hence a primary interest of the current study. It is expected conducting remediation and training in three domains (i.e., cognition, emotion processing, and social cognition) will improve psychosocial functioning to greater extent than is achieved by treating cognition alone.

### Personalization

Given the multifaceted nature of impairment in depression, it is reasonable to assume that deficits will not occur in a uniform nature within individuals. Certain individuals may be disadvantaged specifically in domains of emotional processing, while others are more disadvantaged in terms of cold cognitive ability or social cognition. Previous interventions may not have led to consistent improvements in psychosocial functioning because individual differences in domain-specific impairment were not addressed. In fact, existing interventions assume that group means (i.e., norms) reflect impairment at an individual level, and hence that a generalized treatment approach is sufficient. Given the heterogeneity of impairment observed between individuals with MDD (12), it is possible that a standard treatment approach is not optimal (50). In particular, standard treatment approaches may misappropriate resources and time to clinical domains which are not relevant or helpful to the individual. In contrast, personalized approaches tailor treatment by targeting baseline deficits within individuals, enabling treatment to focus on impaired domains, while also spending less time addressing more functional domains. Personalization may improve treatment efficiency and efficacy, in contrast to the traditional standard approach.

The current investigation will evaluate the personalized approach, by comparing psychosocial functioning following a personalized intervention and a standard (i.e., non-personalized) intervention. The personalized intervention will tailor treatment tasks around baseline individual deficits, such that the individual’s most impaired domains receive the greatest attention. Given the importance of cognition, emotion processing, and social cognition in determining psychosocial functioning, the CERT-D treatment will be tailored by patterns of impairment observed in these three domains. For example, a participant who demonstrates severe cognitive impairment will receive a greater number of treatment sessions devoted to cognition, with fewer sessions devoted to emotion processing and social cognition. Overall, it expected that both personalized and standard treatments will result in improved psychosocial functioning, which is expected to be retained over a 6-month observational period. However, it is predicted that the clinical effect on psychosocial function will be greater following personalized, relative to standard, treatment.

In summary, the cognitive and emotional recovery training program for depression (CERT-D) study will evaluate a novel treatment for depression, employing an integrated cognitive, emotional and social cognitive approach. The primary outcome will be change in psychosocial function over the intervention and subsequent observational period. Performance in several secondary domains will also be evaluated, including depression symptom severity, resilience, occupational functioning, cognitive failures, and functional disability. In addition, the study will examine biomarkers of cognitive and psychosocial dysfunction in depression, providing an opportunity to expand our knowledge of this domain. This research will advance the field by evaluating the efficacy of integrating training of cold cognition with emotion processing and social cognition. The integrated approach contrasts with traditional cognitive remediation, which has primarily focused on the improvement and application of executive training. In addition, the current methods allow evaluation of the relative clinical efficacy of personalized and standard treatment approaches in remediating psychosocial dysfunction in MDD.

## Methods

### Objectives

The primary objective of the CERT-D is to evaluate whether treating cognition, emotion processing, and social cognition leads to immediate and longitudinal benefit in psychosocial functioning. To achieve this objective, participants will complete either a personalized or standard (non-personalized) treatment devoted to increasing performance in these domains. Participants in the personalized group will receive a tailored treatment incorporating a greater number of sessions devoted to their domain(s) of primary baseline dysfunction. By contrast, the standard treatment group will complete a pre-established battery of treatment independent of baseline deficits.

The key hypotheses and aims of the current study are as follows:

*Hypothesis 1*: Overall, psychosocial functioning will be improved at 8 weeks [end of randomized, controlled treatment (RCT)] relative to baseline. Psychosocial functioning in the observational period (post-RCT) will not decline at 3 and 6 months relative to post-RCT baseline.
*Related Aim*: To evaluate which subdomains of psychosocial function (e.g., autonomy, social relationships) are sensitive to change over time from the CERT-D.
*Hypothesis 2*: Performance in secondary outcome measures will improve at 8 weeks relative to baseline, and will be retained over a 6-month observational period. Secondary outcomes include occupational functioning, cognitive failures, functional disability, resilience, and depression symptom severity.
*Hypothesis 3*: It is expected that the personalized treatment group will display greater psychosocial improvement at 8 weeks (compared to baseline) than subjects who complete a standard treatment.
*Hypothesis 4*: Biological and genomic signatures will be associated with psychosocial functioning, as well as cognitive, emotional, and social cognitive performance.

### Study Design and Recruitment

The CERT-D study will commence in July 2017. The study will be a RCT, comparing the clinical efficacy of a personalized and standard intervention. Psychosocial functioning is considered the primary outcome. An effect size of approximately *d* = 0.5 is expected with regards to change in psychosocial function over the intervention. Given the intended sample size of 100, the study will achieve statistical power of 89% (1 − β = 89). The rationale for this “medium” effect size is that previous interventions have found positive outcomes approximate to this magnitude (5, 35), for example in cognitive functioning.

**Figure 1:**
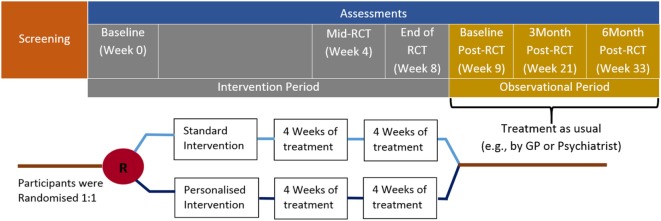
Clinical timeline for the CERT-D. Participants will complete 2 training sessions per week (16 sessions total) over the 8-week intervention period.

The trial will include 16 treatment sessions designed to improve cognition, emotion processing, and social cognition administered over an 8-week period. Assessments will occur at baseline, 4 weeks (mid-RCT), 8 weeks (end of RCT), and in the observational period at baseline (week 9) and 3 and 6 months post-RCT (see Figure 1). Assessments will measure psychosocial functioning, cognition, emotional state, social cognition, as well as occupational functioning, depression symptom severity, functional disability, cognitive failures, and resilience. Three assessment visits will also include taking blood for biomarker and genetic analysis. Recruitment will include individuals between the ages of 18 and 75, who will be invited to participate *via* research clinics of the Department of Psychiatry, University of Adelaide, and within the Central Health Network in Adelaide, South Australia. It could be argued that the age range should be restricted to younger adults (<60), such that subjects with geriatric depression are excluded. However, recent work has suggested that cognitive training strategies are efficacious in the treatment of geriatric depression (47, 51, 52). Given the current intervention shares many components of traditional cognitive training, it is suggested that the CERT-D intervention should benefit older (i.e., 60–75 years) patients.

### Inclusion and Exclusion Criteria

Individuals with mild–moderate MDD according to DSM-IV-TR criteria (17) will be recruited for the current study. Severely depressed subjects will not be included, as the complexity from treatment tasks would likely result high rates of deterrence. Subjects identified through screening with bipolar or anxiety disorders will be excluded, as will subjects with schizophrenia, a learning disorder, eating disorder or a pervasive developmental disorder. Subjects with current brain injury or impairment which could affect cognitive function (e.g., neurodevelopmental disorders, dementia) will be excluded. In addition, subjects will be withdrawn following subsequent severe brain/head injury, development of dementia, psychosis, or development of neurological conditions such as multiple sclerosis or Parkinson’s disease.

### Ethics

The CERT-D has been approved by the Human Research Ethics Committees at the Royal Adelaide Hospital (approval number: R20170611) and The University of Adelaide (approval number: R20170611). All details of participant involvement will be conveyed to study participants both in writing and verbally before informed consent is obtained.

There are no severe adverse effects expected to result from the current treatment. However, participants will be withdrawn from treatment if they demonstrate a significant increase in depression symptom severity [20% increase in Montgomery Asberg Depression Rating Scale (MADRS) score] and referred to their treating psychiatrist or GP.

### Randomization Process

**Figure 2:**
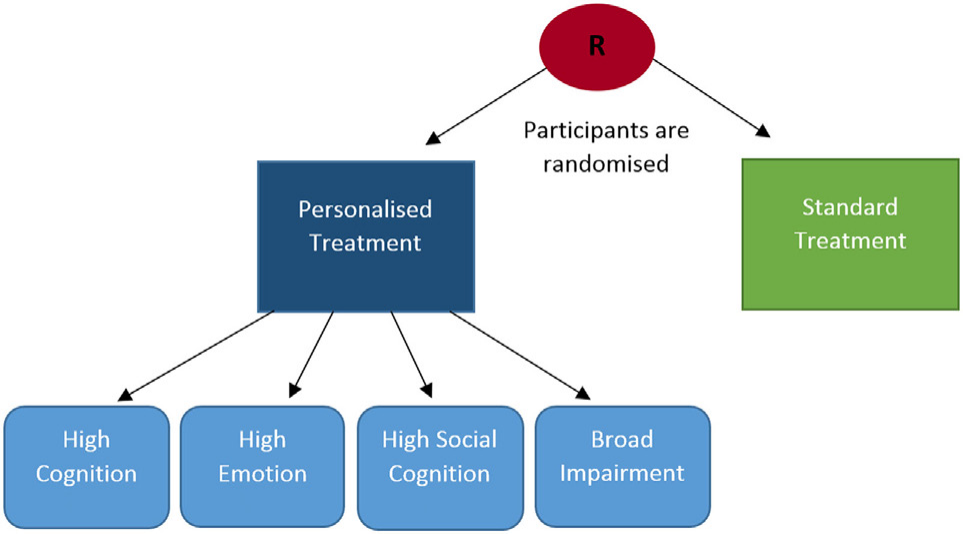
CERT-D randomization process. Several streams of treatment are available for subjects in the personalized group (high cognition, high emotion, high social cognition, broad impairment), whereas treatment is the same for every participant in the standard treatment group.

After screening, subjects will be randomly allocated with computer software to receive either personalized or standard (non-personalized) treatment (see Figure 2). In both the standard and personalized intervention groups each treatment session will consist of domain-specific tasks. Every treatment session will be repeated at least once, to ensure that subjects have the opportunity to practice the treatment tasks.

### Personalized Treatment

Personalized interventions will be tailored to address subjects’ most impaired domains. Domain-specific tests will be administered at baseline to determine the modality and degree of individual impairment. On the basis of baseline deficits, subjects in the personalized group will be allocated to one of four potential treatment arms; (1) high cognition treatment, (2) high emotion treatment, (3) high social cognition treatment, and (4) broad impairment treatment (see Figure 2). Subjects will be allocated to streams 1, 2, or 3 if impairment is primarily represented in one of the three baseline domains. In treatment arms, 1–3 subjects will complete a greater number of intervention sessions devoted to the domain of primary dysfunction. Subjects who are significantly impaired in two or more domains will be allocated to broad impairment treatment. Broad impairment treatments will incorporate a similar number of intervention sessions in each domain, but will be initiated more gradually (i.e., initial treatment sessions will be shorter in duration) and will use simpler tasks.

### Standard Treatment

Subjects allocated to the standard (i.e., non-personalized) intervention will receive an identical intervention regardless of baseline impairment. The standard intervention will be comprised of approximately an equal number of treatment sessions in each domain. Unlike broad impairment treatment, standard treatment sessions will be the full duration from the outset and the difficulty curve of treatment tasks will be steeper.

## Clinical, Self-Report, and Cognitive Assessments

### Screening

Participants will be screened for presence of MDD with the MINI 600 Neuropsychiatric Diagnostic Interview. The MINI is well validated and has demonstrated high specificity and sensitivity, as well as close concordance with the American Psychiatric Association Diagnostic Criteria (SCID), and the Composite International Diagnostic Interview (ICD-10) (53, 54). The MADRS will also be administered as a measure of depression symptom severity (55).

### Assessment Visits (Baseline—6 Months Post-RCT)

In total, six assessments of functioning and performance will occur over the CERT-D timeline (see Figure 1). In the intervention period, subjects will complete assessments at baseline, 4 weeks (mid-RCT), and 8 weeks (end of RCT). The observational period will comprise assessments in week 9 (baseline post-RCT), as well as 3 and 6 months post-RCT.

#### Psychosocial Functioning

Psychosocial functioning will be assessed with the Functioning Assessment Short Test (FAST); a clinician administered 24-item scale. The FAST includes questions which gauge subjects’ abilities across several psychosocial domains of daily living (e.g., autonomy, leisure, financial issues). The FAST takes approximately 6 min to complete (9) and will be administered by a clinician blind to experimental group allocation.

#### Cognitive Functioning

Cognitive functioning will be assessed with the THINC-it tool; a digitally administered screening instrument for cognitive impairment in depression. The THINC-it involves four objective tests of cognitive performance, including choice reaction time, a *1*-back memory task, the Trail Making Test Part B and digit symbol substitution. The THINC-it also includes a 5-item component of the self-reported perceived deficit questionnaire, as an indication of retrospective cognitive dysfunction (56).

#### Emotion

Participants’ emotional state will be assessed with the Positive and Negative Affect Schedule (PANAS), which requires subjects to indicate the intensity of current and recent emotions. Scores are calculated separately for positive and negative emotions, with higher scores indicating greater intensity. The separability of positive and negative mood scores enables discriminate bilateral mood evaluation, which is not possible with unilateral scales or with measures of depression symptom severity alone. The PANAS has well-supported psychometric properties, including high internal consistency and convergent and discriminant validity (57, 58).

#### Social Cognition

The Weschler Adult Intelligence Scale Advanced Clinical Solutions Social Cognition Test (WAIS-IV-ACS) will be used to assess participants’ social cognition. This component of the WAIS was developed in response to the finding that memory for faces and affect recognition was independent of other cognitive abilities, suggesting a unique neuropsychological construct in social cognition (38). The test involves three tasks designed to identify social cognitive impairment: Affect Naming, Prosody-Face Matching, and Prosody-Pair Matching.

#### Depression Symptom Severity

Depression symptom severity will be assessed with the MADRS and the Structured Interview Guide of the Hamilton Anxiety and Depression Scale (SIGH-AD). The SIGH–AD is a 31-item structured interview that combines the Hamilton Depression Scale (HAM-D, 17 items) and the Hamilton Anxiety Scale (HAM-A, 14 items) (59).

#### Work Productivity

Two scales will be used to evaluate occupational functioning: The Endicott Work Productivity Scale (EWPS) and the Work Productivity and Impairment Questionnaire (WPAI). Both the EWPS and WPAI are self-report questionnaires designed to measure occupational productivity (60, 61). Importantly the EWPS measures overall occupational impairment, whereas the WPAI measures the extent to which a particular issue (e.g., depression) negatively affects occupational functioning.

#### Resilience

Resilience will be assessed with “The Resilience Scale” (62). The scale involves completing a 26-item Likert scale, of which each item makes a broad statement measuring the participants’ perceived resilience (e.g., “I usually manage one way or another”). The Resilience Scale has shown high internal consistency and concurrent validity (63), as well as validation with a number of age and ethnic groups (64).

#### Functional Disability

Disability in daily life will be evaluated with the Sheehan Disability Scale (65, 66). This will involve participants self-reporting the extent to which depression symptoms disrupt three domains: (1) work/school, (2) social life, and (3) family life/home responsibilities. The Sheehan disability scale achieves high internal consistency (0.89) and concurrent validity, with high scores are indicative of mental health disorders (65).

#### Cognitive Failures

Cognitive failures can be defined as everyday slips of memory and attention (e.g., forgetting a colleague’s name). The current study will evaluate cognitive failures with the Cognitive Failures Questionnaire (CFQ), which gages the frequency of cognitive failures experienced in the past 6 months. The CFQ appears to have acceptable construct validity, as factor analysis has indicated that CFQ items load primarily to a single construct (67).

#### Blood Specimen

Blood taking will occur at baseline, week 8 (end of RCT), and at 6 months post-RCT. These samples will enable analyses of biomarker associations with psychosocial functioning, as well as the effect of the CERT-D intervention on biomarker levels. Blood serum analyses will include of evaluation of cytokine concentration (e.g., IL-8, TNF), CRP levels, and neurotransmitter activity. Samples will be stored in secure refrigerators with stringent access rights at the Adelaide Health and Medical Sciences building.

## CERT-D Intervention Details

The intervention is comprised of 16 total treatment sessions. Each session will be devoted to one of the underlying domains of psychosocial functioning targeted by the CERT-D (i.e., cognition, emotion processing, social cognition). Tasks completed within sessions will be presented in both digital and pen and paper formats. Several digital tasks will be completed with the psychology experiment building language (PEBL) software (68), which incorporates a number of freely distributed psychological tests presented in a game-like manner. Other treatment tasks will be administered in a pen and paper format.

### Cognition Treatment

Cognition treatment sessions will involve cold cognition tasks, with the expectation that subjects’ performance in these tasks will improve over time. The researcher will emphasize the value of cold cognitive skills in everyday life and discuss transfer of these skills to functional domains (e.g., psychosocial functioning). Each cognition session will focus on one of three cognitive modalities: Executive functioning, visuospatial working memory, and verbal working memory (69). Executive treatment sessions will focus on attention, inhibition, problem solving and mental updating (70). Executive tasks include Berg’s card sorting test, symbol counting, and the Stroop task (68). Visuospatial treatment sessions will focus on spatial learning, mental rotation, and spatial coordination (71), including map learning (72), figure learning, Corsi blocks (73), and matrix rotation tasks (68). Verbal treatment sessions will target verbal sequencing, reading span, and digit span (74). Verbal training will utilize immediate and delayed verbal memory tasks in the SCIP battery (75), and the reading span and digit span tests in PEBL (68).

### Emotion Processing Treatment

The aim of emotion processing sessions will be to address negative emotional biases and cognitive-emotional (i.e., “hot cognition”) impairment in depression. Training tasks will attempt to increase participants’ performance in hot cognitive tasks, in which depressed patients typically underperform (34). In addition, the subject will be encouraged to discuss any cognitive–emotional issues which arise in these training sessions, which the expectation that raising awareness and understanding may help subjects cope with and overcome emotional dysfunction (e.g., emotional avoidance, poor reappraisal) (32). Emotional treatment tasks include emotional brain storming (49), an emotional *n*-back (35), and emotion word list tasks (76). Taken together, these tasks are intended to embed emotional stimuli within traditional cognitive remediation techniques (e.g., the *n*-back task). These strategies are based on the rationale that improving cognitive management and processing of emotional material will reduce negative attentional and cognitive biases in subjects with MDD (35).

### Social Cognition Treatment

Social cognitive training sessions will involve targeting performance in domains of facial affect recognition, prosody detection, body language, and interpersonal communication. Taken together, it is intended that social cognitive treatment will improve subjects’ ability to synthesize a broad spectrum of social information, make clearer theory of mind judgments, and reduce social tension and avoidance. The researcher will emphasize the importance of social cognition in everyday life and encourage participants to exercise acquired social skills outside of treatment sessions. Social cognition training tasks will include a “reading the mind in the eyes” task (77), an interpersonal word list task (78) and theory of mind scenarios (44, 45). These tasks are designed to improve the evaluation of facial affect, verbal tone, and body language, while also highlighting the pitfalls of making unfounded or overly negative assumptions about others’ intentions and emotions.

## Intervention Outcomes, Analyses, and Dissemination

The primary outcome of the CERT-D will be psychosocial functioning. Specifically, it is expected that psychosocial functioning will improve at 8 weeks (end of RCT) relative to baseline. Post-RCT psychosocial functioning is expected to be maintained over the 6 months observational period. Performance in secondary outcome measures is expected to demonstrate a similar effect of treatment. Secondary outcomes include depression symptom severity, occupational functioning, resilience, functional disability, and perceived cognitive failures. In addition, the personalized treatment group is expected to show greater improvement over time relative to the standard treatment group. Finally, the association between biomarkers and psychosocial and cognitive performance before and following treatment will be evaluated.

Statistical analyses will be performed with a mixed model ANOVA with time (baseline, 8 weeks, baseline post-RCT, 3 months post-RCT, 6 months post-RCT) and treatment type (personalized, standard) as independent variables. Psychosocial functioning (FAST score) will be the dependent variable. It is expected that overall psychosocial functioning will improve at post-treatment relative to baseline, and that this improvement will be greater in the personalized treatment arm relative to the standard treatment arm. Biomarker analyses require specifically tailored software and will ultimately be entered into simple and stepwise linear regression analyses. These analyses will determine whether individual biomarkers (e.g., CRP), or combinations of biomarkers, are related to cognition (i.e., THINC-it performance) or psychosocial functioning.

Within the personalized treatment group, further analyses will evaluate whether there are any differences in psychosocial functioning owing to the domain-specific treatment groups. That is, will there be any differences in post-RCT psychosocial functioning between the high cognition, high emotion, and high social cognition treatment groups? Given the novelty of the current treatments, these analyses will be exploratory. As with the primary outcome, analyses for this outcome will be conducted with mixed model ANOVAs.

The anonymized data obtained in the current study will be owned by the University of Adelaide, with access restricted to the research team responsible for the project. The results of the CERT-D study will be published in scientific journals, which will discuss clinical efficacy of personalized and standard approaches in light of the data obtained. The authors will also conduct a public lecture to discuss the broader topic of personalized psychiatry, which will include dissemination of outcomes in the current study.

## Discussion

Existing literature suggests that psychosocial functioning is associated with depression severity and with longitudinal treatment outcomes (2, 3). Mutual interaction between psychosocial domains (e.g., work productivity, socializing) in depressed persons may exacerbate, maintain, or lead to recurrent depression. As an example, poor cognitive functioning may lead to issues maintaining work, which will cause financial strain and lead to reduced social interactions and impaired affect. Given the importance of psychosocial functioning in depression recovery, the development of treatments designed to target psychosocial functioning is justified.

Current research by the Baune group suggests that cognitive, emotional, and social cognitive domains underpin psychosocial functioning (12, 16). The CERT-D will target these domains with repeated training sessions over an 8-week intervention period. Cognitive and cognitive–emotional treatment approaches for depression have received empirical support (2, 32, 35). Initial research suggests treating social cognition may also be beneficial (16, 79, 80); however, further evidence is needed to establish the efficacy of treating social cognitive functioning in depression. The CERT-D study addresses this gap in knowledge by evaluating a treatment approach integrating all three underlying domains of psychosocial functioning (i.e., cognition, emotion processing, and social cognition). Treatment benefits of the CERT-D are intended to transfer to occupational functioning, resilience, functional abilities, and cognitive performance. Reinforcing improved performance and positive interaction between these domains is expected to develop a framework which encourages and rewards reduction of depression symptoms.

The directionality of recovery in the CERT-D is divergent from traditional depression therapies. That is, existing therapies (e.g., psychotherapy) target reduction in negative depression symptoms with the expectation that improvements in functional and psychosocial domains will follow. By contrast, the current treatment targets psychosocial and functional improvements from the outset. Importantly, the CERT-D integrates treatment across cognitive, emotional, and social domains rather than focusing on one of these domains alone. Integrating treatment across multiple domains is intended to counteract negative feedback between impaired domains which may occur in more selective treatments.

A possible disadvantage of administering training across three domains is that treatment benefits to cold cognitive functioning (e.g., executive functioning) will be attenuated in comparison to traditional cognitive remediation. Reduced executive gains in the CERT-D treatment could result from the equal emphasis given to social, emotional, and cognitive domains, as opposed to focusing purely on cold cognition. However, we do not anticipate this issue to be a crucial issue for two key reasons: (1) emotional and social–cognitive training demand similar cognitive effort and are equally complex in comparison to traditional cognitive training. As a result, emotional and social training may also benefit executive functioning. (2) Individuals who demonstrate significantly impaired cold cognition will be allocated to the “High Cognition” program within the personalized arm. This treatment program emphasizes cognitive treatment above emotional and social domains, and will hence evaluate the efficacy of predominantly cognitive training, relative to training all three domains in the “standard” treatment group. If cognition training alone is identified as having greater clinical efficacy than the integrated training approach, then this finding will also contribute to the value of the current study.

In summary, the CERT-D will make three primary contributions: (1) the evaluation of a novel psychological treatment for depression. Psychosocial function is considered the primary outcome, and is expected to be improved by integrated treatment of underlying domains. (2) The comparison of the relative clinical efficacy of personalized and standard treatment approaches. Taking baseline deficits into account may enable more efficient and effective psychosocial recovery, as impaired domains receive greater attention. In combination, contributions (1) and (2) may lead to the development of an intervention which could be conducted in parallel or in lieu of other treatments for depression. (3) The current study will also improve our understanding of the genomic, neurological and biological correlates of psychosocial and cognitive dysfunction in depression. Taken together, these findings will advance the field of personalized psychiatry by evaluating the relative efficacy of a standard and personalized treatment approach, in addition to testing the overall value of targeting psychosocial deficits in MDD.

## Ethics Statement

This study will be carried out in accordance with the recommendations of the NHMRC national statement on ethical conduct in human research, Royal Adelaide Hospital HREC with written informed consent from all subjects. All subjects will give written informed consent in accordance with the Declaration of Helsinki. The protocol was approved by the Royal Adelaide Hospital HREC and the University of Adelaide HREC.

## Author Contributions

BB: primary investigator. BB is involved in study design, recruitment, supervision, and in his capacity as study Doctor. MK: research officer. MK is involved in study design, recruitment, and administering the CERT-D intervention and assessments.

## Conflict of Interest Statement

The authors declare that the research was conducted in the absence of any commercial or financial relationships that could be construed as a potential conflict of interest. The reviewer MJ and handling editor declared their shared affiliation.
